# Greater self-efficacy, psychological readiness and return to sport amongst paediatric patients compared with adolescents and young adults, 8 and 12 months after ACL reconstruction

**DOI:** 10.1007/s00167-023-07623-5

**Published:** 2023-10-20

**Authors:** Baldur Thorolfsson, Ramana Piussi, Thorkell Snaebjornsson, Jon Karlsson, Kristian Samuelsson, Susanne Beischer, Roland Thomeé, Eric Hamrin Senorski

**Affiliations:** 1 Department of Orthopaedics, Sahlgrenska University Hospital, Gothenburg, Sweden; 2 Department of Orthopaedics, Institute of Clinical Sciences, Sahlgrenska Academy, University of Gothenburg, Gothenburg, Sweden; 3 Sahlgrenska Sports Medicine Center, Gothenburg, Sweden; 4 Department of Health and Rehabilitation, Institute of Neuroscience and Physiology, Sahlgrenska Academy, University of Gothenburg, Gothenburg, Sweden; 5 Sportrehab Sports Medicine Clinic, Gothenburg, Sweden; 6 Department of Orthopaedics, Sahlgrenska University Hospital, 43180 Mölndal, Sweden

**Keywords:** Anterior cruciate ligament, ACL, Paediatric, Adolescent, Registry

## Abstract

**Purpose:**

The purpose of this study was to evaluate differences in rehabilitation-specific outcomes between paediatric patients, adolescents and young adults within the first 2 years after anterior cruciate ligament (ACL) reconstruction. A further aim was to determine whether patient age was associated with an increased risk of not achieving symmetrical muscle function within the first 2 years after ACL reconstruction.

**Methods:**

The patient data in the present study were extracted from the rehabilitation outcome registry, Project ACL. Patients aged 11–25 years registered for primary ACL reconstruction with a hamstring tendon autograft between April 1, 2013 and November 23, 2020 were included. A total of 691 patients met the inclusion criteria and were included in the study; 41 paediatric patients (females 11–13, males 11–15 years), 347 adolescents (females 14–19, males 16–19 years) and 303 young adults (females 20–25, males 20–25 years).

**Results:**

The comparison between groups revealed that 70% of paediatric patients, 39% of adolescents and 35% of young adults had returned to knee-strenuous sport at 8 months and that 90% of paediatric patients, 71% of adolescents and 62% of young adults had returned to sport at 12 months. Paediatric patients also reported higher scores compared with both the other patient groups on the Knee Self-Efficacy Scale (K-SES) and the Anterior Cruciate Ligament Return to Sport after Injury scale (ACL-RSI) at 8 and 12 months.

**Conclusions:**

A larger proportion of paediatric patients had returned to sport compared with adolescents and young adults 8 and 12 months after ACL reconstruction. Paediatric patients also reported higher self-efficacy and greater psychological readiness to return to sport at 8 and 12 months than the other two groups. No differences in terms of muscle function tests when comparing paediatric patients, adolescents and young adults were found.

**Level of evidence:**

II.

## Introduction

Young patients have been reported to have a high rate of return to sport (RTS) after anterior cruciate ligament (ACL) reconstruction [3, 16, 19]; however, early RTS has also been associated with a high rate of graft rupture and contralateral ACL injury [16]. Adolescents have reported a lower rate of acceptable knee function after ACL reconstruction and run an increased risk of sustaining a second ACL injury compared with their older counterparts [26, 27]. In addition, adolescents have previously been reported to return to knee-strenuous sports earlier than young adults, without having recovered acceptable knee function after ACL reconstruction [3]. Previous studies have also shown that only a minority of younger athletes regain symmetrical muscle function in both strength and hop tests after ACL reconstruction before return to sport [3, 28].

Well-planned and completed rehabilitation is essential for regaining muscle strength and function after ACL reconstruction and previous studies have found that symmetrical muscle function is a potential protective factor against a second ACL injury [10, 15].

Several studies have reported the results in hop tests and strength tests among adults recovering after ACL reconstruction [8, 20, 22, 29], but few, if any, have focused on the results in hop and strength tests in association with knee-related self-efficacy and RTS among children and adolescents recovering after ACL reconstruction.

The aim of this study was to evaluate differences in rehabilitation-specific outcomes between paediatric patients, adolescents and young adults treated with ACL reconstruction within the first two years after the reconstruction. A further aim was to determine whether patient age at the time of ACL reconstruction was associated with an increased risk of not achieving symmetrical muscle function within the first 2 years after an ACL reconstruction.

## Materials and methods

### Ethical considerations

Patients received written information on Project ACL and informed consent was obtained before participation. Participation was completely voluntary for patients. The extracted data are confidential and patient sex and age can only be identified by authorised personnel from the patient’s social security number. Project ACL has ethical approval from the Swedish Ethical Review Authority (registration number: 2020–02501).

### Patient data

The patient data in this study were extracted from the rehabilitation outcome registry, Project ACL. Project ACL is a collaboration between the University of Gothenburg, Sahlgrenska University Hospital and the Swedish National Knee Ligament Registry (SNKLR) [1]. The registry includes patients from the western part of Sweden and was established in September 2014 [3]. The registry consists of two parts: a battery of validated patient-reported outcome measurements (PROMs) and a battery of validated muscle function tests for hop performance and leg-muscle strength [1]. Patients in Project ACL are regularly evaluated first at 10 weeks and thereafter at 4, 8, 12, 18 and 24 months after the ACL injury or reconstruction. In terms of hop performance and leg-muscle strength, isokinetic concentric strength testing of knee extension and knee flexion is started at 10 weeks and hop performance testing is started at 4 months [1, 3, 13, 21]. For the present study, data from the 8-, 12-, 18- and 24-month follow-ups were extracted and used.

### Patient-reported outcome measurements

The patients in Project ACL answer a series of validated PROMs at each follow-up. They include the Anterior Cruciate Ligament Return to Sport after Injury scale (ACL-RSI), the European Quality 5 Dimensions 3 levels (EQ5D-3L), the Knee injury and Osteoarthritis Outcome Score (KOOS), the Knee Self-Efficacy Scale (K-SES), the Physical Activity Scale (PAS) and the Tegner Activity Scale (Tegner). In this study, answers to the ACL-RSI, K-SES and Tegner were extracted and used for analysis.

The ACL-RSI evaluates the confidence, emotion and risk appraisal in relation to RTS after an ACL injury [21]. Each item is graded from 1 to 10, where 10 is the most positive response. The total score for the 12-item version therefore ranges from 10 to 120, where a score of 120 indicates the highest confidence, most positive emotions and lowest risk appraisal in relation to RTS [30]. The ACL-RSI has demonstrated good validity, internal consistency (Cronbach’s alpha = 0.948), low floor and ceiling effects and high construct validity when evaluated against the K-SES, KOOS and ACL-Quality of Life scales [17]. In this study, the validated 12-item Swedish version was used.

The K-SES was first introduced in 2006 and it evaluates perceived knee-related self-efficacy in patients who suffer an ACL injury [24]. The original K-SES is comprised of 22 items and is divided into two subscales: present knee self-efficacy and future knee self-efficacy [24]. Each item is graded from 0 to 10, with 10 being the most positive response representing the greatest belief in carrying out a given physical task. The results for each question are added and the sum is divided by the number of questions, generating a mean value ranging from 0–10. In this study, we used the modified 18-item version, the K-SES18, which has been reported to have acceptable validity and reliability in assessing knee-efficacy in patients, 16–50 years of age, after ACL reconstruction [4].

The Tegner was first presented in 1985 and is widely used as a tool to grade knee-strenuous activity, particularly in patients with knee injuries [23]. The scale has previously been validated for ACL patients showing acceptable test–retest reliability (Intraclass Correlation Coefficient (ICC) = 0.8) and acceptable floor and ceiling effects [5]. The scale ranges from 0 to 10, based on the level of intensity, where 0 represents sick leave due to knee injury, 1 represents the least possible knee-strenuous activity and 10 represents international elite level in sports such as football and rugby. Level 6 on the Tegner is equivalent to participation in knee-strenuous sport and is referred to as return to sports (RTS) in the present study.

### Muscle function tests

The assessments of muscle strength and hop ability were supervised by registered physiotherapists trained in the standardised test procedure [1]. An isokinetic concentric strength test of knee extension and knee flexion was performed using a Biodex System 4 (Biodex Medical Systems, Shirley, New York). After strength testing, three hop tests were performed in the following order: vertical hop, hop for distance and side-hop test. The test procedure, including a warm-up, familiarisation with sub-maximum practice trials, rest and maximum testing, has previously been described in detail [1, 3, 13]. For all tests, the best of three attempts was registered in the database, with the exception of the side hop, for which a single 30-s attempt was allowed.

### Patients

**Fig. 1:**
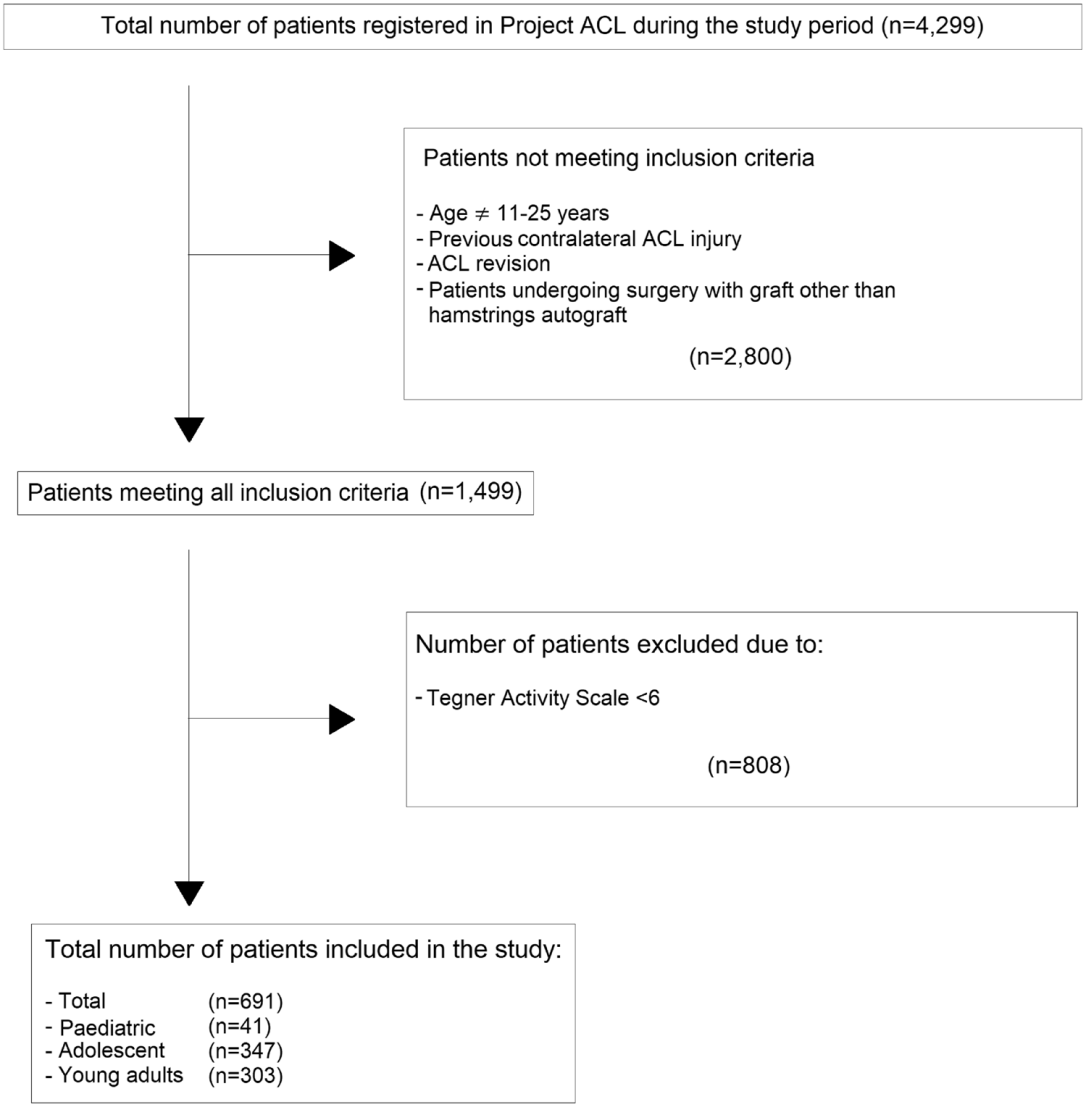
Flow chart of patients meeting inclusion and exclusion criteria. *ACL* anterior cruciate ligament

Patients aged 11–25 years registered for primary ACL reconstruction with a hamstring tendon autograft between April 1, 2013 and November 23, 2020 in Project ACL were eligible for inclusion. Only patients who underwent primary ACL reconstruction and had undergone no previous knee surgery were included in the study. Patients who reported an activity level lower than six on the Tegner were excluded. Figure 1 presents a flow chart of inclusion and exclusion criteria.

The cohort was stratified into age groups of females, 11–13, 14–19 and 20–25 years respectively, and males, 11–15, 16–19 and 20–25 years respectively, as shown in Table 1. The stratification into different age groups was performed to create one group of skeletally immature individuals, a second group of individuals who underwent ACL reconstruction at or just after the time of physeal closure and a third reference group of skeletally mature young adults. Unfortunately, radiographs are not kept in the registry and the age of skeletal maturity was therefore set at 14 years in females and 16 years in males, as this is generally considered a fair estimation [7, 9, 11].

**Table 1:** Definition of age groups in the study

	Female	Male
Paediatric	11–13 years*	11–15 years*
Adolescents	14–19 years*	16–19 years*
Young adults	20–25 years	20–25 years

*The age of skeletal maturity was set at 14 years in females and 16 years in males

### Variables and outcome

The following data were extracted from Project ACL; patient age at index surgery, sex, weight, height, Tegner score before and after surgery and the results of the K-SES and ACL-RSI questionnaires after surgery, as well as the results of the muscle function tests. Symmetrical muscle function was defined as achieving a Limb Symmetry Index of ≥ 90% (LSI 90) in all five tests of muscle function. The LSI 90 is based on the recommendation from the European Board of Sports Rehabilitation [25]. This cut-off is commonly used to evaluate patients after ACL reconstruction, because achieving an LSI 90 has been reported to reduce the risk of subsequent ACL injury after returning to sport [10, 18]. The primary study outcome was patients returning to sport, i.e. achieving level six or higher on the Tegner, as well as patients achieving an LSI 90 on all 5 muscle function tests, 8, 12, 18 and 24 months after the ACL reconstruction.

### Statistical analysis

Statistical analysis was performed using the SAS statistical analysis system (SAS/STAT, version 9.4; SAS Institute Inc., Cary, NC, USA). For categorical variables, count (n) and proportion (%) were presented. For continuous variables, the mean and standard deviations (SD) and the median with the first and third quartile were presented. For comparisons between groups, Fisher’s exact test (lowest 1-sided p-value multiplied by 2) was used for dichotomous variables and Fisher’s non-parametric permutation test was used for continuous variables. The confidence interval for the mean difference between groups is based on Fisher’s non-parametric permutation test. The significance level for all statistical analyses was set at 5%.

## Results

During the study period, a total of 4299 patients with an ACL injury were registered in Project ACL. Of these, 691 patients met the final inclusion criteria: 41 paediatric patients (mean age 14.7 ± 1.0 years), 347 adolescents (mean age 17.1 ± 1.5 years) and 303 young adults (mean age 23.0 ± 1.7 years). The demographic characteristics of the study groups are presented in Table 2. The total number of patients at each follow-up time point is presented in Table 3.

**Table 2:** Demographic data of the study groups showing means, standard deviations, medians, range and interquartile ranges

	Paediatric (n = 41)	Adolescents (n = 347)	Young adults (n = 303)
Age at reconstruction (years)	14.7 (1.0) 15 (12; 15) (14.3; 15.0)	17.1 (1.5) 17.2 (14; 19) (17.0; 17.3)	23.0 (1.7) 23 (20; 25) (22.8; 23.2)
Height (cm)	175.3 (10.5) 177 (140; 197) (172.0; 178.6)	171.7 (8.6) 171 (151; 200) (170.8; 172.6)	176.3 (9.2) 177 (154; 200) (175.3; 177.4)
Weight (kg)	65.4 (12.2) 66 (35; 95) (61.4; 69.3)	66.5 (10.1) 65 (47; 105) (65.5; 67.6)	75.4 (12.3) 75 (49; 117) (74.0; 76.7)
BMI (kg/m^2^)	22.0 (6.4) 21.1 (15.8; 59.2) (20.0; 24.0)	22.5 (2.4) 22.3 (17.3; 32.7) (22.3; 22.8)	24.1 (2.7) 24.1 (17.5; 34.6) (23.8; 24.4)
Male	32 (78.0%)	100 (28.8%)	173 (57.1%)
Time to return to knee-strenuous activity [months] (Tegner ≥ 6)	9.8 (4.1) 8.1 (2.1; 23.9) (8.5; 11.1)	10.7 (5.8) 10.0 (2.1; 36.3) (10.0; 11.4)	9.9 (6.1) 8.2 (0.9; 36.3) (9.0; 10.8)
Time from injury to surgery [months]	4.8 (7.1) 3.1 (0.2; 46.3) (2.5; 7.0)	5.6 (8.2) 3.4 (0.3; 90.6) (4.7; 6.5)	8.2 (10.7) 4.8 (0.2; 74.9) (7.0; 9.4)

*BMI* Body Mass Index, *cm* centimetres, *kg* kilograms, *n* number, *Tegner* Tegner Activity Scale

**Table 3:** Number of patients at each follow-up

	Paediatric	Adolescents	Young adults
8 months	n = 34	n = 298	n = 239
12 months	n = 33	n = 269	n = 202
18 months	n = 26	n = 200	n = 138
24 months	n = 20	n = 145	n = 113

*n* number of patients

### Patient-reported outcome measurements

A significantly larger proportion of paediatric patients had returned to sport (achieved level six or higher on the Tegner) compared with adolescents and young adults at 8 and 12 months. The comparison between groups revealed that 70% of paediatric patients, 39% of adolescents and 35% of young adults had had returned to knee-strenuous sport at 8 months, while 90% of paediatric patients, 71% of adolescents and 62% of young adults had RTS at 12 months. Paediatric patients also reported significantly higher scores compared with both other patient groups on the K-SES and the ACL-RSI at 8 and 12 months. At 8 months, the paediatric patients had a mean K-SES score of 8.8 ± 0.8 compared with 8.2 ± 1.3 among adolescents and 7.9 ± 1.5 among young adults. At 12 months, the mean K-SES scores were 9.3 ± 0.7, 8.7 ± 1.1 and 8.5 ± 1.4 points respectively. On the ACL-RSI, the paediatric patients had a mean score of 91.3 ± 16.5 points, adolescents 71.0 ± 22.0 and young adults 66.5 ± 23.9 at eight months. At 12 months, the mean ACL-RSI score was 96.9 ± 17.5, 76.4 ± 24.9 and 72.6 ± 25.7 points respectively. A detailed comparison between the patient groups is presented in Tables 4, 5, 6 and 7.

**Table 4:** Comparison between the study groups at the 8-month follow-up

	Paediatric	Adolescents	Young adults	p-value	p-value	p-value
	(n = 34)	(n = 298)	(n = 239)	Paediatric vs. Adolescents	Paediatric vs. young adults	Adolescents vs. young adults
Tegner Activity Scale						
	7 (2; 10)	5 (2; 10)	4 (1; 10)	0.0060	0.0010	0.015
	n = 30	n = 273	n = 221			
Tegner Activity Scale ≥ 6						
Yes	19 (70.4%)	97 (38.8%)	71 (34.8%)	0.0033	0.0009	n.s
K-SES	8.8 (0.8)	8.2 (1.3)	7.9 (1.5)			
	9 (6.9; 10)	8.4 (2.8; 10)	8.2 (0; 10)			
	(8.5; 9.12)	(8.0; 8.3)	(7.7; 8.1)			
	n = 29	n = 271	n = 221	0.0030	0.0010	n.s
ACL-RSI (10–120)	91.3 (16.5)	71.0 (22.0)	66.5 (23.9)			
	90 (62; 120)	72 (12; 119)	66 (12; 120)			
	(84.8; 97.9)	(68.2; 73.8)	(63.1; 69.9)			
	n = 27	n = 243	n = 192	0.0010	0.0010	0.037
LSI distance hop	96.6 (7.5)	93.9 (9.1)	92.4 (10.4)			
	97.5 (78.1; 110.5)	94.8 (60.8; 118.3)	93.2 (53.6; 121.9)			
	(93.7; 99.5)	(92.7; 95.0)	(90.9; 93.9)			
	n = 28	n = 238	n = 181	n.s	0.032	n.s
LSI vertical hop	96.3 (12.7)	90.2 (16.0)	87.6 (14.5)			
	99.3 (66.5; 116)	90.9 (39.2; 131.3)	89.7 (44.4; 142.7)			
	(91.2; 101.3)	(88.2; 92.2)	(85.4; 89.7)			
	n = 27	n = 241	n = 182	n.s	0.0030	n.s
LSI side hop	99.3 (13.9)	94.6 (18.4)	92.5 (15.5)			
	98.2 (76.5; 130.8)	95.9 (37; 166.7)	93.2 (44; 162.5)			
	(93.6; 104.9)	(92.2; 97.1)	(90.1; 94.8)			
	n = 26	n = 219	n = 170	n.s	0.039	n.s
LSI quadriceps	98.8 (6.5)	96.0 (11.0)	92.5 (10.6)			
	99.1 (86.3; 111.8)	96.3 (48.5; 135.3)	93.8 (47.5; 122.9)			
	(96.3; 101.3)	(94.6; 97.3)	(91.1; 94.0)			
	n = 28	n = 265	n = 202	n.s	0.0040	0.0010
LSI hamstrings	99.6 (14.1)	97.2 (12.7)	95.3 (12.6)			
	101.1 (72.7; 123.5)	97.4 (55.8; 136.4)	96.4 (43.4; 135.3)			
	(94.1; 105.1)	(95.6; 98.7)	(93.6; 97.1)			
	n = 28	n = 264	n = 202	n.s	n.s	n.s
LSI ≥ 90 for all five tests						
Yes	9 (32.1%)	54 (21.4%)	32 (16.5%)	n.s	n.s	n.s

Data are presented as the means with standard deviations, medians with range and interquartile ranges

*ACL-RSI* anterior cruciate ligament return to sport after injury scale, *K-SES* knee self-efficacy scale, *LSI* limb symmetry index, *n.s.* Non-Significant p-value

**Table 5:** Comparison between the study groups at the 12-month follow-up

	Paediatric	Adolescents	Young adults	p-value	p-value	p-value
	(n = 33)	(n = 269)	(n = 202)	Paediatric vs. adolescents	Paediatric vs. young adults	Adolescents vs. young adults
Tegner Activity Scale						
	9 (5; 10)	7 (1; 10)	7 (1; 10)	0.0030	0.0010	n.s
	n = 31	n = 245	n = 176			
Tegner Activity Scale ≥ 6						
Yes	27 (90.0%)	153 (70.5%)	97 (61.8%)	0.0031	0.0009	n.s
K-SES	9.3 (0.7)	8.7 (1.1)	8.5 (1.4)			
	9.4 (7.5; 10)	9 (3.4; 10)	8.8 (3.4; 10)			
	(9.1; 9.6)	(8.6; 8.9)	(8.2; 8.7)			
	n = 31	n = 243	n = 175	0.0010	0.0010	n.s
ACL-RSI (10–120)	96.9 (17.5)	76.4 (24.9)	72.6 (25.7)			
	98.5 (62; 120)	76 (17; 120)	74 (12; 120)			
	(90.1; 103.7)	(73.0; 79.7)	(68.6; 76.6)			
	n = 28	n = 220	n = 160	0.0010	0.0004	n.s
LSI distance hop	97.5 (8.8)	96.8 (7.6)	95.0 (8.9)			
	98.9 (83.5; 119.8)	97.1 (72; 116.1)	94.8 (54.3; 134.2)			
	(94.0; 101.1)	(95.8; 97.8)	(93.5; 96.5)			
	n = 26	n = 207	n = 143	n.s	0.032	n.s
LSI vertical hop	104.2 (15.9)	94.5 (13.3)	92.4 (14.5)			
	104.2 (79.8; 153.1)	95.3 (60; 148.8)	92.9 (54.1; 141.9)			
	(97.6; 110.7)	(92.7; 96.4)	(90.0; 94.8)			
	n = 25	n = 205	n = 143	0.020	0.0004	n.s
LSI side hop	105.1 (17.8)	100.3 (15.5)	96.8 (17.5)			
	101.9 (75; 165)	98.4 (50; 160)	96.6 (46.7; 171.4)			
	(97.8; 112.4)	(98.2; 102.5)	(93.8; 99.7)			
	n = 25	n = 201	n = 140	n.s	0.034	n.s
LSI quadriceps	101.2 (6.9)	99.9 (9.7)	95.6 (10.8)			
	100.7 (85.2; 119.6)	99.2 (71.1; 154.8)	96.9 (52.3; 138.1)			
	(98.4; 104.0)	(98.6; 101.2)	(93.9; 97.4)			
	n = 26	n = 219	n = 153	n.s	0.0100	0.0010
LSI hamstrings	100.7 (13.7)	98.8 (11.1)	97.3 (11.9)			
	98.1 (73.3; 129.6)	100 (58.8; 130.7)	97.2 (51.7; 129)			
	(95.2; 106.3)	(97.3; 100.3)	(95.4; 99.2)			
	n = 26	n = 219	n = 153	n.s	n.s	n.s
LSI ≥ 90 for all five tests						
Yes	12 (48.0%)	78 (37.1%)	43 (29.1%)	n.s	n.s	n.s

Data are presented as the means with standard deviations, medians with range and interquartile ranges

*ACL-RSI* anterior cruciate ligament return to sport after injury scale, *K-SES* knee self-efficacy scale, *LSI* limb symmetry index, *n.s.* Non-Significant p-value

**Table 6:** Comparison between the study groups at the 18-month follow-up

	Paediatric	Adolescents	Young adults	p-value	p-value	p-value
	(n = 26)	(n = 200)	(n = 138)	Paediatric vs. adolescents	Paediatric vs. young adults	Adolescents vs. young adults
Tegner Activity Scale						
	9 (7; 10)	8 (2; 10)	7 (1; 10)	0.0020	0.0004	0.0060
	n = 21	n = 163	n = 115			
Tegner Activity Scale ≥ 6						
Yes	21 (100.0%)	130 (83.3%)	76 (72.4%)	0.0057	0.0047	0.049
K-SES	9.6 (0.4)	9.0 (1.12)	8.8 (1.2)			
	9.6 (8.6; 10)	9.4 (2.7; 10)	9.2 (4; 10)			
	(9.4; 9.8)	(8.8; 9.1)	(8.6; 9.0)			
	n = 21	n = 161	n = 114	0.0016	0.0008	n.s
ACL-RSI (10–120)	102.0 (17.3)	81.4 (26.9)	80.1 (26.8)			
	106 (62; 120)	87 (12; 120)	79 (12; 120)			
	(94.1; 109.9)	(77.0; 85.8)	(74.9; 85.3)			
	n = 21	n = 147	n = 103	0.0012	0.0004	n.s
LSI distance hop	100.5 (7.0)	98.0 (7.9)	95.9 (10.0)			
	100.3 (82.2; 114.5)	98 (67.6; 116.8)	96.8 (48.6; 119.4)			
	(96.8; 104.2)	(96.6; 99.4)	(93.7; 98.2)			
	n = 16	n = 126	n = 80	n.s	n.s	n.s
LSI vertical hop	98.3 (7.6)	98.2 (13.0)	92.9 (14.9)			
	96.5 (86.1; 111.9)	96.9 (56.6; 134.1)	94 (50.3; 129.9)			
	(94.4; 102.3)	(95.9; 100.6)	(89.5; 96.2)			
	n = 17	n = 121	n = 79	n.s	n.s	0.0088
LSI side hop	111.5 (27.1)	99.8 (13.6)	97.0 (11.7)			
	103 (93; 200)	100 (56.3; 182.6)	98.6 (58.3; 122.6)			
	(97.1; 125.9)	(97.4; 102.2)	(94.3; 99.6)			
	n = 16	n = 126	n = 78	0.021	0.0034	n.s
LSI quadriceps	102.8 (10.1)	100.7 (9.2)	99.5 (10.6)			
	100 (91; 127)	100.9 (59; 125)	98.8 (63.8; 131.5)			
	(97.6; 107.9)	(99.1; 102.2)	(97.2; 101.7)			
	n = 17	n = 132	n = 87	n.s	n.s	n.s
LSI hamstrings	100.1 (12.7)	99.8 (11.1)	99.0 (11.9)			
	98.7 (79.4; 128.3)	100 (65.8; 129.9)	101 (67.3; 132.4)			
	(93.5; 106.6)	(97.9; 101.7)	(96.4; 101.5)			
	n = 17	n = 132	n = 87	n.s	n.s	n.s
LSI ≥ 90 for all five tests						
Yes	10 (62.5%)	59 (46.1%)	55 (66.3%)	n.s	n.s	n.s

Data are presented as the means with standard deviations, medians with range and interquartile ranges

*ACL-RSI* anterior cruciate ligament return to sport after injury scale, *K-SES* knee self-efficacy scale, *LSI* limb symmetry index, *n.s.* Non-Significant p-value

**Table 7:** Comparison between the study groups at the 24-month follow-up

	Paediatric	Adolescents	Young adults	p-value	p-value	p-value
	(n = 20)	(n = 145)	(n = 113)	Paediatric vs. adolescents	Paediatric vs. young adults	Adolescents vs. young adults
Tegner Activity Scale						
	9 (7; 10)	8 (2; 10)	7 (1; 10)	0.0024	0.0004	0.0092
	n = 14	n = 111	n = 89			
Tegner Activity Scale ≥ 6						
Yes	14 (100.0%)	85 (83.3%)	55 (66.3%)	n.s	0.011	0.012
K-SES	9.5 (0.6)	9.1 (1.0)	8.5 (1.8)			
	9.9 (8.4; 10)	9.5 (4.8; 10)	9.2 (0.4; 10)			
	(9.2; 9.9)	(8.9; 9.3)	(8.2; 8.9)			
	n = 14	n = 107	n = 89	n.s	0.010	0.0024
ACL-RSI (10–120)	104.2 (18.0)	88.1 (27.2)	74.1 (31.2)			
	112.5 (65; 120)	101 (14; 120)	75.5 (12; 120)			
	(93.8; 114.6)	(82.8; 93.4)	(67.3; 80.8)			
	n = 14	n = 103	n = 84	0.026	0.0004	0.0012
LSI distance hop	102.8 (8.4)	98.1 (8.4)	98.1 (6.8)			
	103.3 (82.8; 119.8)	97.8 (73.9; 118.9)	97.9 (81.8; 116.7)			
	(98.1; 107.4)	(96.3; 100.0)	(96.2; 100.0)			
	n = 15	n = 81	n = 53	n.s	0.027	n.s
LSI vertical hop	97.4 (11.5)	98.4 (13.2)	96.4 (12.3)			
	96.4 (78.9; 114)	96.8 (57.1; 141.2)	96.2 (71.5; 124.7)			
	(91.0; 103.8)	(95.4; 101.4)	(92.9; 99.8)			
	n = 15	n = 76	n = 52	n.s	n.s	n.s
LSI side hop	105.4 (10.9)	101.3 (14.2)	97.9 (10.7)			
	101.4 (92.3; 128.9)	101.8 (46.2; 133.3)	100 (64.5; 118.5)			
	(99.3; 111.4)	(98.1; 104.5)	(94.8; 100.9)			
	n = 15	n = 78	n = 50	n.s	0.018	n.s
LSI quadriceps	99.8 (6.8)	100.9 (10.4)	99.6 (8.9)			
	99.3 (87; 116)	101.2 (60.8; 121.3)	100.4 (76.2; 118.1)			
	(96.0; 103.5)	(98.7; 103.2)	(97.3; 101.9)			
	n = 15	n = 84	n = 61	n.s	n.s	n.s
LSI hamstrings	94.4 (10.0)	99.3 (12.2)	99.2 (11.3)			
	94.5 (75.7; 107.1)	98.2 (74.5; 143.3)	99.2 (68.7; 121.8)			
	(88.8; 99.9)	(96.6; 101.9)	(96.3; 102.1)			
	n = 15	n = 84	n = 61	n.s	n.s	n.s
LSI ≥ 90 for all five tests						
Yes	6 (40.0%)	40 (50.6%)	19 (37.3%)	n.s	n.s	n.s

Data are presented as the means with standard deviations, medians with range and interquartile ranges

*ACL-RSI* anterior cruciate ligament return to sport after injury scale, *K-SES* knee self-efficacy scale, *LSI* limb symmetry index, *n.s.* Non-Significant p-value

### Symmetrical muscle function

**Fig. 2:**
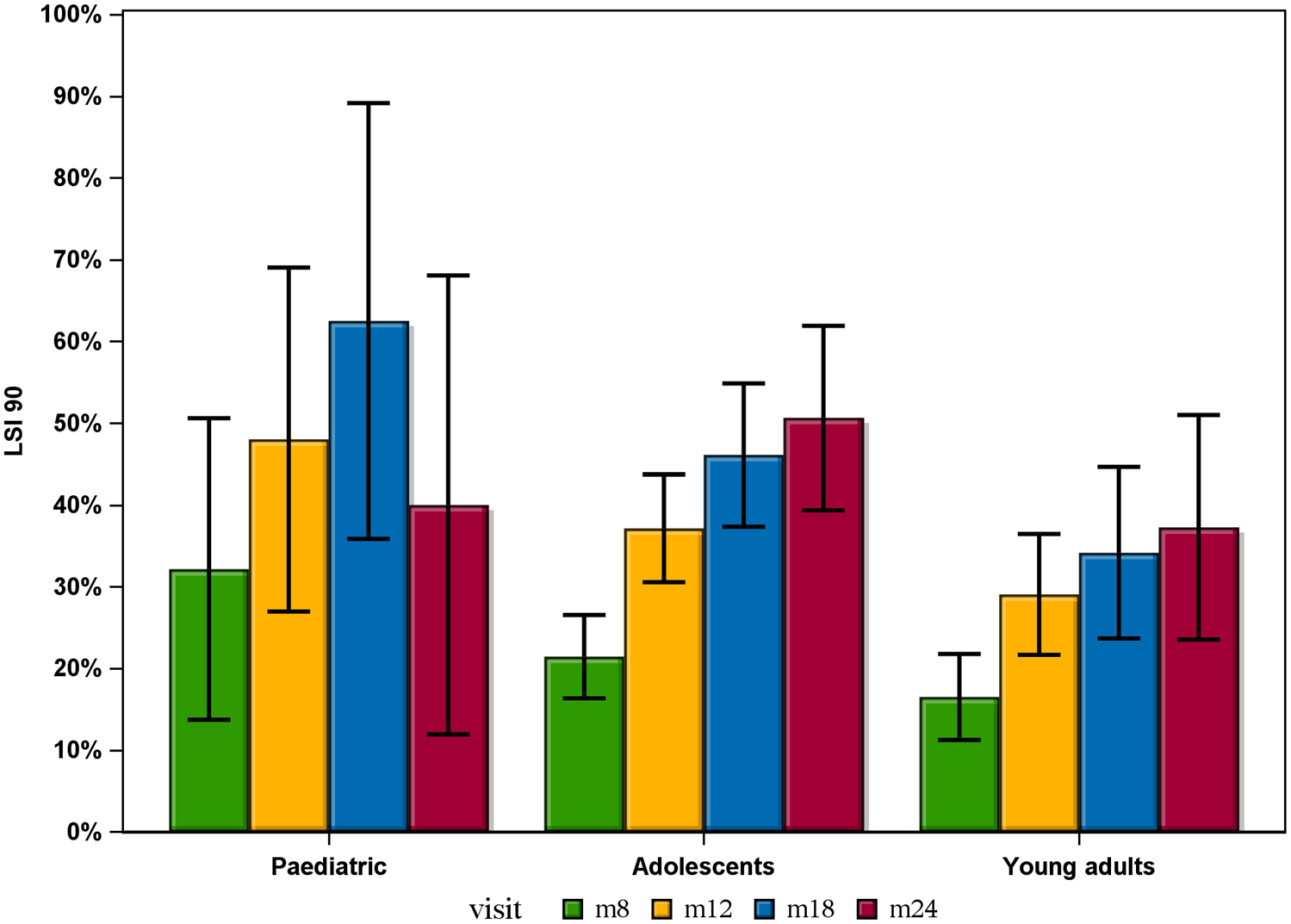
Bar chart showing the proportion of patients achieving an LSI 90 at each visit and error bars indicating the 95% confidence interval

**Fig. 3:**
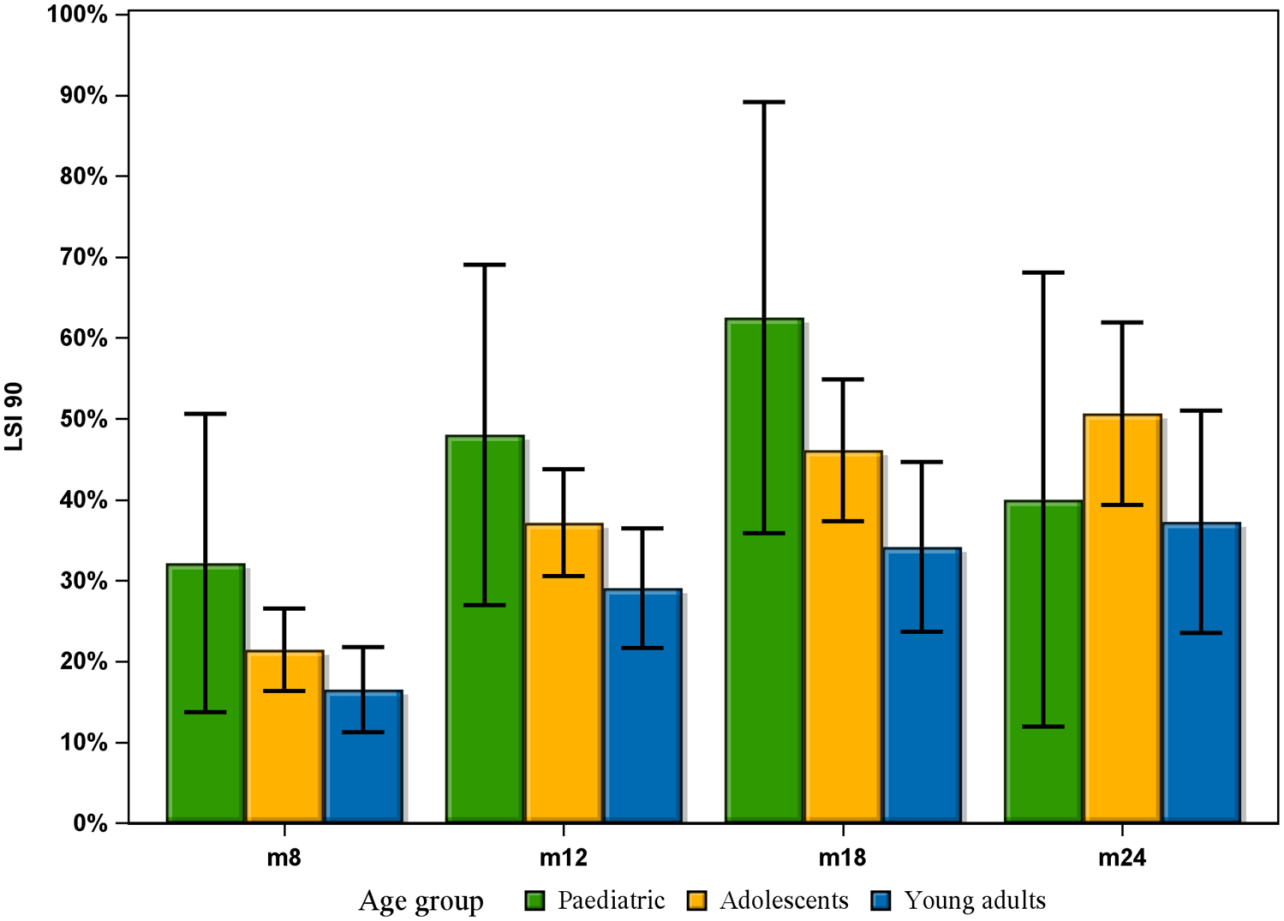
Bar chart showing each visit and the proportion of each patient group achieving an LSI 90. Error bars indicate the 95% confidence interval

No significant differences were seen between any of the groups at any follow-up visits, in terms of achieving an LSI 90 for all five muscle function tests. Each group had an increasing proportion of patients achieving an LSI 90 at each follow-up time point, with increasing time from ACL reconstruction, apart from paediatric patients where a smaller proportion achieved an LSI 90 at the 24-months follow-up compared with the 18-months follow-up. Paediatric patients had the largest proportion of patients achieving an LSI 90 at the 8-, 12- and 18-month follow-ups, followed by adolescents and young adults with the smallest proportion of patients achieving an LSI 90. However, these differences did not reach statistical significance. A comparison between the groups in terms of an LSI 90 at the different follow-ups can be seen in Figs. 2 and 3.

## Discussion

The main finding in this study was that a significantly larger proportion of paediatric patients had returned to sport (achieved level six or higher on the Tegner) compared with adolescents and young adults at 8 and 12 months. The comparison between groups revealed that 70% of paediatric patients, 39% of adolescents and 35% of young adults had returned to knee-strenuous sport at 8 months, while 90% of paediatric patients, 71% of adolescents and 62% of young adults had done so at 12 months. The scientific literature contains few previous studies that compare the time to RTS after ACL reconstruction between paediatric patients, adolescents and young adults. However, some previous studies have reported results similar to ours; i.e. that younger patients tend to return earlier to knee-strenuous sport compared with young adults [2, 3, 14]. In one of these studies, also based on data from Project ACL, Beischer et al. [3] reported that 50% of adolescents (15–20 years) had returned to knee-strenuous sport 8 months after ACL reconstruction compared with 38% of young adults (21–30 years) and, at 12 months, the proportions were 74% and 63% respectively. These findings are similar to the findings in the present study, where 39% of adolescents and 35% of young adults had returned to knee-strenuous sport at 8 months and 71% of adolescents and 62% of young adults had RTS at 12 months. The difference in RTS can possibly be explained by the different age grouping in the studies. The present study also revealed that 70% of paediatric patients had RTS at 8 months and 90% of the same group had RTS at 12 months, which is significantly higher than in the other two age groups. Cordasco et al. [6] previously reported similar results from an American cohort of patients under the age of 20. In that study, 92% of skeletally immature patients undergoing ACL reconstruction returned to the same level of sport within 2 years.

The paediatric patients reported significantly higher scores than both the other patient groups on the K-SES and the ACL-RSI, indicating higher self-efficacy and greater psychological readiness to return to sport at 8 and 12 months. A similar pattern was seen at the 18- and 24-month follow-ups, but it was not statistically significant between all groups at each visit. At 8 months, the paediatric patients had a mean K-SES score of 8.8 compared with 8.2 among adolescents and 7.9 among young adults. At 12 months, the mean K-SES score was 9.3, 8.7 and 8.5 points respectively. On the ACL-RSI, the paediatric patients had a mean score of 91.3 points, adolescents 71.0 and young adults 66.5 at 8 months. At 12 months, the mean ACL-RSI score was 91.3, 71.0 and 72.6 points respectively. Similar to the results of the present study, previous studies have shown that younger patients report higher scores on the ACL-RSI compared with adults [32]. Younger patients have previously also been shown to be more content than adults with their knee function 1 year after ACL reconstruction [12], but the opposite findings have also been reported 2, 5 and 10 years after ACL reconstruction [26]. A recent study from the same patient registry showed that patients who had greater psychological readiness, i.e. greater confidence in performance, fewer negative emotions and lower risk appraisal in relation to RTS, were more likely to suffer an ACL re-rupture [21]. These findings therefore raise the question of whether children and adolescents return to sport too early after ACL reconstruction because of their high psychological readiness to RTS.

No significant differences were seen between any of the groups at any follow-up time points, in terms of achieving an LSI 90 on all five muscle function tests. Each group contained an increasing proportion of patients achieving an LSI 90 at each follow-up, with increasing time from the ACL reconstruction, apart from paediatric patients where a smaller proportion achieved an LSI 90 at the 24-months follow-up compared with the 18-months follow-up. An interesting trend was observed where the paediatric patients had the largest proportion of patients achieving an LSI 90 at the 8-, 12- and 18-month follow-ups, followed by adolescents and young adults with the smallest proportion of patients achieving an LSI 90. However, this difference was not statistically significant. Hamrin Senorski et al. [13] have previously published results from the same patient registry showing that fewer than one in four patients had achieved an LSI 90 one year after ACL reconstruction. Although the patient cohort in that study had a mean age of 26.7 years, it resembles the findings in the present study where 29.1% of young adults had achieved an LSI 90 at 1 year. As previously mentioned, achieving an LSI 90 has been reported to reduce the risk of subsequent ACL injury after returning to sport [10, 18]. It can therefore be questioned whether or not achieving more than 110% strength or hop ability compared with the uninjured knee is a positive result, as this was seen in some of the patients in this study.

In 2017, Toole et al. published the results from an American cohort of young patients aged 14–22, where only 13.9% had achieved an LSI 90 on all strength and hop tests at the time of RTS, which took place on average just over 8 months from ACL reconstruction [28]. The present study shows substantial differences, where 32.1% of paediatric patients, 21.4% of adolescents and 16.5% of young adults had achieved an LSI 90 on all muscle function tests at 8 months.

Although the patient-reported outcomes and muscle function tests commonly used to evaluate patients after ACL reconstruction provide valuable information, several other factors can play a role in whether or not young athletes return to their previous level of sport. In a study from 2017, where Webster et al. [31] studied the RTS among patients with an ACL reconstruction under the age of 20, the most common reason cited by the group of patients who never returned to their preinjury sport was fear of re-injury. However, the second most common reason was work or study commitments, which accounted for as much as 30%.

The main limitation of this study is that different patient cohorts are included at each follow-up. Another limitation is that data related to sports participation in Project ACL, reported by patients on the Tegner, only reflect how knee strenuous the sport in which the patients participated actually was and when the patients returned to their sport. Data for sport exposure, i.e. whether the patients participated in modified or unrestricted training or competition, or the frequency of participation, were not available. Individual radiographs are unfortunately not included in the registry. Instead skeletal maturity was generalised depending on age, which may have caused some individuals to fall into the wrong category. A-priori sample size calculation was not performed and we are aware that some comparisons are underpowered due to the limited number of patients available at each follow-up. The uneven sex distribution at different follow-ups can be regarded as a limitation. Lastly, neither the ACL-RSI nor the K-SES has been validated for paediatric patients.

This is the first study to investigate differences in RTS, subjective knee function and muscle function between paediatric patients, adolescents and young adults after a primary ACL reconstruction. The patient-reported outcomes that were used, as well as the methods for assessing strength and hop performance, have good to acceptable psychometric properties for evaluating adult patients after ACL reconstruction [3], but they have not been validated for paediatric patients.

## Conclusions

Seventy per cent of paediatric patients, 39% of adolescents and 35% of young adults had RTS at 8 months, while 90% of paediatric patients, 71% of adolescents and 62% of young adults had RTS at 12 months. Paediatric patients reported greater self-efficacy and psychological readiness to return to sport at 8 and 12 months than the other two groups. No differences were found in terms of muscle function tests when comparing paediatric patients, adolescents and young adults.

## Data Availability

All data used to support the findings of this study are available upon request baldur.thorolfsson@gu.se.

## Abbreviations

ACL: Anterior cruciate ligament
ACL-RSI: Anterior cruciate ligament return to sport after injury scale
BMI: Body Mass Index
CI: Confidence interval
CM: Centimetres
EQ5D-3L: European Quality 5 Dimensions 3 levels
ICC: Intraclass Correlation Coefficient
KG: Kilograms
KOOS: Knee injury and Osteoarthritis Outcome Score
K-SES: Knee Self-Efficacy Scale
K-SES18: The modified 18-item version of the Knee Self-Efficacy Scale
LSI 90: Limb Symmetry Index of ≥ 90%
n: Count
PAS: Physical activity scale
PROMs: Patient-reported outcome measurements
RTS: Return to sport
SD: Standard Deviation
SNKLR: Swedish National Knee Ligament Registry
Tegner: Tegner Activity Scale
